# The Application of UAV-Based Hyperspectral Imaging to Estimate Crop Traits in Maize Inbred Lines

**DOI:** 10.34133/2021/9890745

**Published:** 2021-04-10

**Authors:** Meiyan Shu, Mengyuan Shen, Jinyu Zuo, Pengfei Yin, Min Wang, Ziwen Xie, Jihua Tang, Ruili Wang, Baoguo Li, Xiaohong Yang, Yuntao Ma

**Affiliations:** ^1^College of Land Science and Technology, China Agricultural University, Beijing 100193, China; ^2^State Key Laboratory of Plant Physiology and Biochemistry, National Maize Improvement Center of China, China Agricultural University, Beijing 100193, China; ^3^College of Agronomy, Henan Agricultural University, Zhengzhou 450002, China; ^4^Agricultural Artificial Intelligence and Crop Phenotype Engineering Research Center, Inner Mongolia Institute of Biotechnology, Huhhot 010070, China

## Abstract

Crop traits such as aboveground biomass (AGB), total leaf area (TLA), leaf chlorophyll content (LCC), and thousand kernel weight (TWK) are important indices in maize breeding. How to extract multiple crop traits at the same time is helpful to improve the efficiency of breeding. Compared with digital and multispectral images, the advantages of high spatial and spectral resolution of hyperspectral images derived from unmanned aerial vehicle (UAV) are expected to accurately estimate the similar traits among breeding materials. This study is aimed at exploring the feasibility of estimating AGB, TLA, SPAD value, and TWK using UAV hyperspectral images and at determining the optimal models for facilitating the process of selecting advanced varieties. The successive projection algorithm (SPA) and competitive adaptive reweighted sampling (CARS) were used to screen sensitive bands for the maize traits. Partial least squares (PLS) and random forest (RF) algorithms were used to estimate the maize traits. The results can be summarized as follows: The sensitive bands for various traits were mainly concentrated in the near-red and red-edge regions. The sensitive bands screened by CARS were more abundant than those screened by SPA. For AGB, TLA, and SPAD value, the optimal combination was the CARS-PLS method. Regarding the TWK, the optimal combination was the CARS-RF method. Compared with the model built by RF, the model built by PLS was more stable. This study provides guiding significance and practical value for main trait estimation of maize inbred lines by UAV hyperspectral images at the plot level.

## 1. Introduction

Maize has the largest yield and the widest planting area among any other crops in the world [1–3]. Total leaf area (TLA, defined as the sum of all leaf area of a single plant) and leaf chlorophyll content (LCC) are closely related to crop photosynthesis and transpiration [4–6]. Many researches showed that SPAD value could well represent the relative value of leaf chlorophyll content, which can be used to quickly diagnose nitrogen status of crop in the field [7, 8]. Aboveground biomass (AGB, defined as the total amount of organic matter of plant aboveground per unit area) plays an important role in the utilization of light energy and the formation of dry matter [9, 10]. Thousand kernel weight (TWK, defined as the weight of a thousand grains) is an indicator of grain size and fullness in breeding and also an important basis for field yield prediction [11, 12]. Therefore, monitoring of TLA, SPAD value, AGB, and TWK can scientifically and efficiently provide evidence for evaluating crop growth and predicting grain yields. Traditional phenotypic analysis is laborious, inefficient, and unable to meet the needs of high-throughput screening for crop breeding [13]. Unmanned aerial vehicles (UAVs) provide a new way to analyze biophysical traits fast, economically, and nondestructively with high-spatial and temporal resolution images.

UAV imaging technology has become one of the important techniques to obtain high-throughput phenotypic traits in breeding and has been widely used in wheat [14], maize [15], rice [16], sorghum [17], soybean [18], sugar-beet [19], potatoes [20], etc. Initially, UAV digital images were firstly applied in extracting crop phenotypic traits, including emergence rate [21], canopy coverage [22–24], leaf area index [4, 5], and aboveground biomass [25–27]. Due to the lack of near-infrared bands more related to crop nutritional activity, it is difficult to achieve high-precision monitoring of crop nutritional traits, such as canopy chlorophyll content and canopy nitrogen content. Compared with digital images, UAV multispectral images have near infrared or red edge bands and have been widely used in chlorophyll, nitrogen, and other related traits [28]. However, due to the wide band of multispectral images, most of them have been applied in field crops under different cultivars, nitrogen application rates, and planting densities [16, 17, 19, 28]. In crop breeding, there are small differences of phenotypic traits among varieties, which cannot be reflected by digital and multispectral images. The UAV hyperspectral image provides spectrums per pixel and has a unique advantage in capturing subtle feature information [29–31]. However, there are few reports on the application of UAV hyperspectral images to hundreds of breeding materials. The purpose of this study is to analyze the potential of UAV hyperspectral images in high-throughput estimation of phenotypic traits of maize inbred lines at the plot level.

UAV hyperspectral images have its advantages of narrower spectrum band and high spatial resolution for the extraction of weak phenotypic traits in crop breeding. The high autocorrelation between narrow bands will inevitably lead to data redundancy. Therefore, before building the estimation model, it is necessary to screen the sensitive bands of target features or mathematically transform the spectral data [32]. Correlation analysis is the most common method used to screen sensitive bands, but it cannot solve the autocorrelation problem of adjacent bands [33]. Principal component analysis (PCA) is commonly used in hyperspectral images. The newly formed principal component information often obscures the unique characteristic bands of the target objects, thus affects the universality of the model [34]. SPA is a forward-loop variable selection method that is widely used in spectral technology. SPA can effectively extract information from severely overlapped spectral information to minimize the effect of collinearity between spectral variables [35, 36]. CARS is a feature selection method that can filter out redundant information. CARS has been applied to the selection of sensitive bands for nitrogen content and soluble solid content [37, 38]. This study attempted to compare these two screening methods of sensitive band in monitoring phenotypic traits of maize inbred lines by UAV-hyperspectral imaging.

At present, the applications of UAV hyperspectral image in breeding are relatively few, which concentrate on the single trait of crops, such as yield [39] and dry matter yield [40]. The objectives of this study were to explore the feasibility of estimating main traits of maize inbred lines using UAV-based hyperspectral images of grain filling stage, including AGB, TLA, SPAD value, and TWK. Firstly, the sensitive bands of these traits were selected by the SPA and CARS algorithms. Then, models were constructed using the PLS and RF methods by combining all the bands or the bands screened by SPA and CARS. Finally, the optimal combination was obtained for predicting AGB, TLA, SPAD value, and TWK in maize.

## 2. Materials and Methods

### 2.1. Experimental Materials and Field Measurements

**Figure 1:**
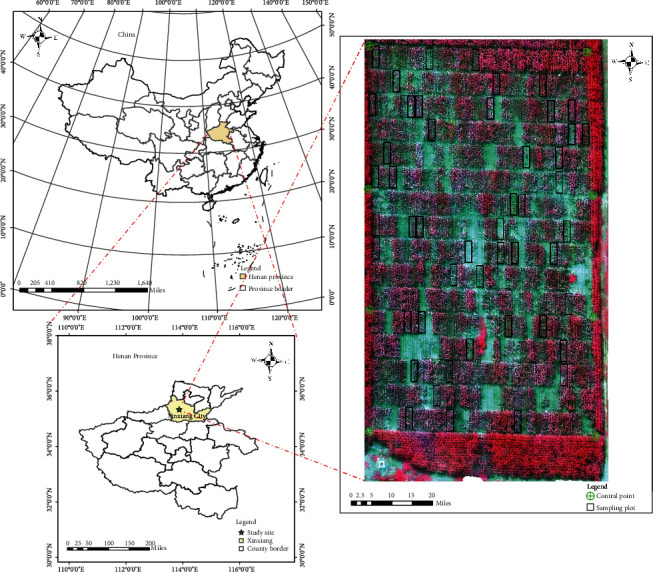
Geographical location of the experimental site.

The field experiment was conducted in Yuanyang Base of Henan Agricultural University, Yuanyang County, Xinxiang City, Henan Province (113.36~114.15 E, 34.55~35.11 N) (Figure 1). Yuanyang County has abundant light and heat, fertile soil, and superior agricultural climate resources. The soil type is mainly tide soil. The annual average temperature is 14°C, and the annual average precipitation is approximately 573 mm.

A set of 498 maize inbred lines with extensive genetic diversity were used as the study materials. According to the genetic background differences, these maize materials were divided into four groups, including tropical, hard rod, nonhard rod, and mixed materials. The mixed materials accounted for no more than 60% of the three bloodlines [41, 42].

The experimental area was approximately 100 m from north to south and 70 m from east to west. Zheng 58 was used as a reference inbred line, which was planted every 50 samples. Each plot contained only one genotype material, and the size of each plot was 1.8 m × 5 m. The row width was 60 cm, and plant spacing was 50 cm. The maize was sowed manually on June 24, 2019. Fertilization and field management were performed in accordance with local management.

Before sowing, 11 ground control points were evenly arranged in the field to obtain accurate geographical positioning. The Huashan T8 intelligent RTK system (CHCNAV–T8, Shanghai, China) was used to locate ground control points accurately. The plane precision of RTK is ± (8 + 1 × 10 − 6 × D) mm, and the elevation accuracy is ± (15 + 1 × 10 − 6 × D) mm. The ground control points were marked with tiles with a size of 30 cm × 30 cm.

In total, 50 representative materials were selected for the study according to genetic differences. Representative plants were selected from the plot on September 22 for destructive sampling. Maize was in the grain filling stage at this time. The grain filling stage is the stage of maize grain formation and is the main period that determines the number of grains. SPAD-502 instrument was used to measure the upper 1/3, middle 1/3, and lower 1/3 of maize ear leaves and its upper and lower leaves. The average value was taken as the SPAD value of this plot. Subsequently, maize stems, leaves, and ears were separated. Leaves were placed neatly on black cloth according to the leaf positions. A SONY ILCE -6300 digital camera (Sony Corporation, Tokyo, Japan) was used to take photos for calculating the TLA of individual plant later. Then, these leaves of each sample were placed into a paper bag. The samples were killed at 105°C for 30 min and dried at 85°C to a constant weight (approximately 24 h). The dry weight of each organ was measured, and the AGB of the sample was obtained. In total, 20 kinds of maize were harvested from 50 species sampled on October 24, 2019. The maize cob was threshed, dried, and weighed to calculate the TKW. Finally, there are 48 effective samples on AGB, TLA, and SPAD value and 20 samples on TWK.

### 2.2. Unmanned Aerial Vehicle and Camera Setup

**Figure 2:**
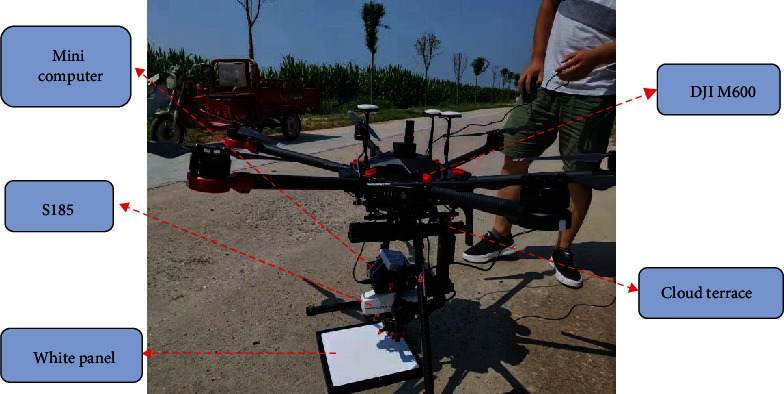
UAV-based hyperspectral imaging system.

On September 22, 2019, the weather was sunny, windless, and cloudless. At approximately 12 : 00, hyperspectral images of maize were obtained by a DJI Matrice 600 Pro Hexacopter (DJI, Shenzhen, China) equipped with a Cubert UHD185 hyperspectral imaging spectrometer (Cubert GmbH, Ulm, Baden Württemberg, Germany).  Figure 2 shows the UAV-based hyperspectral imaging system, which mainly included the DJI M600, cloud terrace, minicomputer, UHD 185 hyperspectral imager, and reference whiteboard. The UHD 185 hyperspectral imager has the characteristics of full-frame, nonscanning, and real-time imaging. The spectral range of the sensor is 450-950 nm, and the spatial resolution is 4 nm, with 125 bands. Before the drone took off, the dark current of the UHD 185 camera was corrected by a microcomputer. The reference whiteboard was used for radiometric calibration. The surface reflectance of the ground object was obtained directly during flight. The forward overlap of the image was 85%, and the lateral overlap was 80%. The fight altitude aboveground level was 60 m, and the flight speed was approximately 6 m/s.

### 2.3. Image Processing and Data Extraction

Based on obtained photos of maize leaves, calculation of TLA was performed (Python 3.0). Firstly, the EXG (Excess green index (EXG)) was used to extract green leaves from background. Then, a photo containing only maize leaves was obtained after binarization, noise removal (gauss filter), and contour extraction (canny edge detect operators). Finally, the cumulative sum of the area that included pixels of maize leaves was calculated to obtain the TLA.

The collected panchromatic and hyperspectral images were spliced and corrected, and the plot canopy spectrum was extracted. In this study, image stitching was carried out with the CubertPilot software from the Cubert Company in Germany and the Agisoft PhotoScan software developed by Agisoft, LLC. Each hyperspectral image and corresponding panchromatic image were fused using the Cubert-Pilot software to obtain the fused hyperspectral image. Then, the Agisoft PhotoScan software was used to complete the stitching of the hyperspectral image based on the point cloud data of the panchromatic image. Finally, a hyperspectral image with 125 bands was obtained, and the ground resolution is 1 cm.

Hyperspectral data of maize were obtained by removing the soil background in the plot by *k*-means cluster analysis in ENVI 5.3 (Esri Inc., Redlands, USA). The clustering criterion of the *k*-means algorithm is minimizing the sum of the squares of the distance from the pixel to the center of the class. The basic idea is iteratively moving all the centers one by one until the convergence criterion is satisfied. The overall classification accuracy was 94.75%, which met the research requirements.

The vector file of each plot was obtained in the Arcgis 10.6 (Esri Inc., Redlands, USA) software. To avoid the mutual influence of the plot, the vector file of the plot size was 4.4 mm × 1.2 m. The average value of the maize spectral reflectance in each vector region was calculated using IDL language as the maize canopy spectrum of the plot.

### 2.4. Data Analysis and Modeling

**Figure 3:**
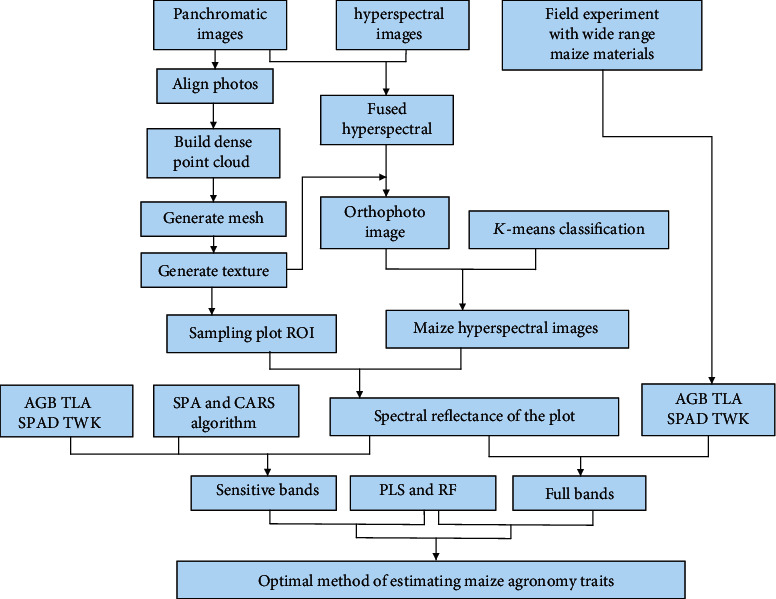
Main flow chart of the research.

In this paper, the SPA and CARS algorithms were used to select sensitive bands for various traits. Partial least square regression and random forest regression were used to compare the model results for the AGB, TLA, SPAD, and yield of maize. Screening of the bands was performed with the Matlab R2018a software (MathWorks, Natick, USA). Statistical analysis, modeling, and figure drawings were realized with R 3.5.3 (R Development Core Team, 2019). The schematic diagram of the research is shown in Figure 3.

#### 2.4.1. Selecting Predictor Variables

The spectral wavelengths of hyperspectral data are continuous. The similarity between adjacent wavelengths is very high. There is a large amount of data redundancy, which will affect the timeliness and accuracy of multivariate analysis. Therefore, it is particularly important to select the characteristic variables that can fully represent all the wavelength information.

SPA is a variable selection method that can extract effective information from a large amount of spectral information and minimize the collinear influence between spectral variables [43]. In recent years, researchers have used SPA to select effective wavelengths and achieved good results for crop nutrition [44], quality [45], soil [46], disease [47], etc. The root mean square error (RMSE) was used as the evaluation criterion to determine the final optimal band.

CARS is a variable selection method that imitates the principle of the Darwinian evolution theory “survival of the fittest” [48]. The optimal variable set is finally determined by an adaptive reweighted sampling technique, exponential decay function, and ten-fold interactive test. The selected variable set with interactive verification has the minimum root mean square error, which can filter out redundant information variables.

#### 2.4.2. The Models

In recent years, machine learning has been used popularly with the development of computer technology. Studies have shown that machine learning can usually better deal with strong nonlinear relationships between biophysical and biochemical traits and reflection spectra than traditional regression analysis [49]. In this paper, two widely used data analysis methods were selected to compare and analyze their accuracy of estimating the AGB, TLA, SPAD value, and TWK in maize.

PLS regression is a multivariate statistical regression method. This method was firstly proposed by Geladi and applied to data analysis [50]. PLS regression effectively combines multiple linear regression, principal component analysis, and correlation analysis, which can better eliminate the multiple collinearities among variables and solve the problem of independent variables outnumbering sample numbers.

RF regression is a kind of data analysis and statistical method widely used in machine learning based on multidecision tree classification [51]. The core of the algorithm uses the bootstrap method to carry out simple random sampling from the original sample set to generate a training sample set. This method has strong adaptability to the target data set, good antinoise performance, and strong fitting ability [49, 52].

For determination of AGB, TLA, and SPAD value, the 48 samples were randomly divided into a training set and test set with a split ratio of 7 : 3, and 10-fold cross validation was used to train and optimize the models. Leave-one-out (LOO) cross validation is cumbersome to calculate. However, its sample utilization rate is the highest than other verification methods, which is suitable for small samples. There were only 20 samples for the TWK. Therefore, the TWK prediction adopted leave-one-out cross validation to the construct model. The accuracy of the model was evaluated by three indices: the coefficient of determination (*R*^2^), RMSE, and mean absolute error (MAE). *R*^2^ was used to represent the fitting effect between the simulated value and the measured value. The closer *R*^2^ is to 1, the higher the fitting accuracy of the model. The calculation formula is as follows.

(1) $$\begin{array}{c} R2=1‐\frac{\sum_{i=1}^{n}{\left(y_{i}-\overset{\wedge }{y_{i}}\right)}^{2}}{\sum_{i=1}^{n}{\left(y_{i}-\bar{y_{i}}\right)}^{2}}. \end{array}$$

The RMSE can reflect the degree of deviation between the simulated value and the measured value. The smaller the RMSE value, the higher the fitting accuracy of the estimated model. The calculation formula is as follows.

(2) $$\begin{array}{c} \text{RMSE}=\sqrt{\frac{\sum_{i=1}^{n}{\left(y_{i}-\overset{\wedge }{y_{i}}\right)}^{2}}{n}}. \end{array}$$

The MAE is the average value of the absolute error, which can better reflect the actual situation of the predicted value error. The calculation formula is as follows.

(3) $$\begin{array}{c} \text{MAE}=\frac{\sum_{i=1}^{n}\left|y_{i}-\overset{\wedge }{y_{i}}\right|}{n}. \end{array}$$

Here, ${\hat{y}}_{i}$ is the predicted value, $\bar{y}$ is the mean of the observed values, *y*_*i*_ is the observed value, and *n* is the number of samples.

## 3. Results

Table 1 shows the basic statistics of the measured AGB, TLA, SPAD value, and TWK for maize. The coefficient of variation (CV) of each variable is relatively large, indicating that the phenotypic traits of different genotypes of maize materials are significant different.

### 3.1. The Correlations between Main Traits and the Hyperspectrum

**Figure 4:**
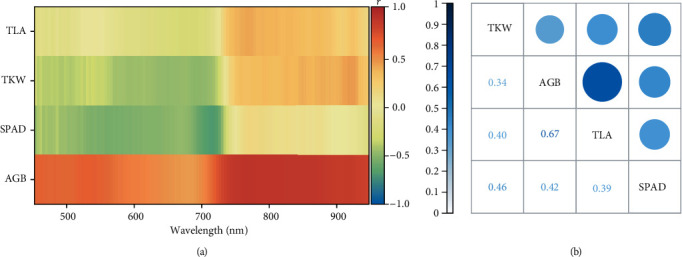
Correlation diagram between the traits and the hyperspectrum (a) and the correlation diagram among traits (b).

Figure 4(a) shows the correlations between AGB, TLA, SPAD value, TWK, and spectral reflectance. The correlation coefficients among AGB, TLA, SPAD value, TWK, and the spectrum were 0.43-0.87, -0.26-0.4, -0.69-0.14, and -0.5-0.43, respectively (Figure 4(b)). To obtain the optimal method for predicting TWK, we comparatively analyzed the correlations among the AGB, TLA, and SPAD value and TWK. It is found that the SPAD value had the highest correlation with TWK. The correlation coefficient was 0.46, which is poor compared with the spectrum. Therefore, the following analysis on TWK is based on hyperspectral data.

### 3.2. Estimation of Maize Aboveground Biomass

**Figure 5:**
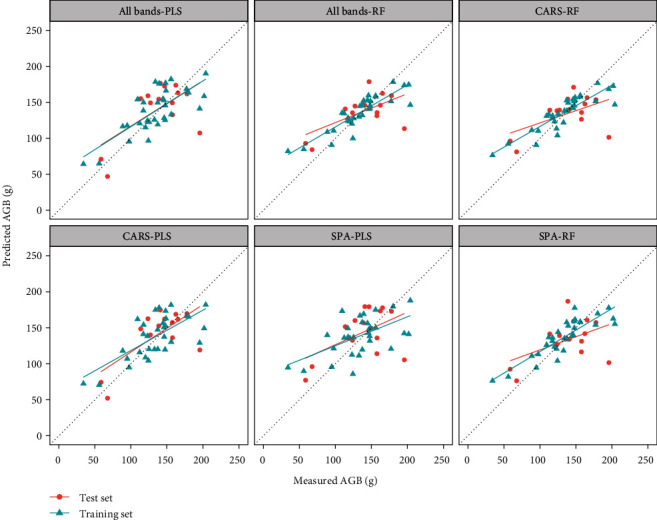
Aboveground biomass (AGB) prediction using different band combinations and the PLS or RF model.

The sensitive bands for AGB were screened by the SPA and CARS algorithms (Table 2). The correlation coefficients between 718 nm and 770 nm and AGB were -0.48 and 0.33, respectively. The CARS algorithm selected a wider range of bands. The bands selected by SPA and CARS and all the bands were used to estimate AGB. Table 2 lists the results of the AGB model constructed by the PLS and RF methods.  Figure 5 shows a scatter plot of the predicted AGB and the measured AGB.

Both the PLS and RF models achieved relatively stable results using all-band modeling. When modeling with the band selected by SPA, both the PLS and RF regression models of the training sets had better results than the test sets. When modeling with the band selected by CARS, the PLS regression models had a higher accuracy and stability compared to the models obtained by RF. Through comparative analysis, we determined that the optimal method for AGB estimation was the CARS-PLS method.

### 3.3. Estimation of Maize Total Leaf Area

**Figure 6:**
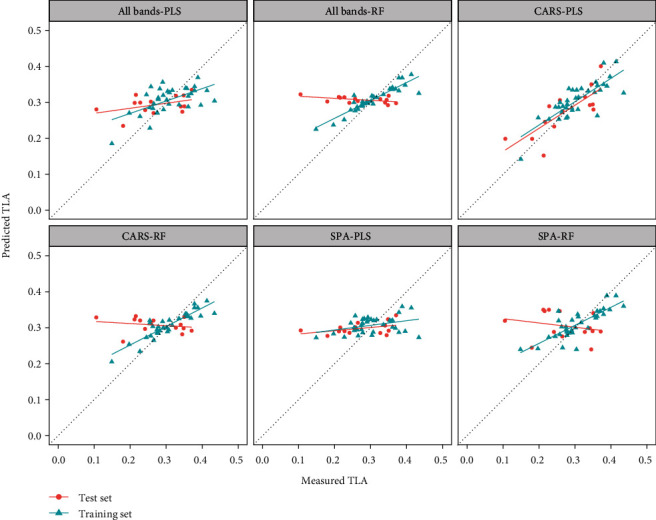
Total leaf area (TLA) prediction using different band combinations and the PLS or RF model.

The sensitive bands for TLA were screened by the SPA and CARS algorithms (Table 3). The band selected by SPA was 770 nm in near-red light. The correlation coefficient between 770 nm and TLA was 0.4, and this band had the highest correlation with TLA among all the bands.  Figure 6 shows a scatter plot of the predicted TLA and the measured TLA.

When using all the bands or the band screened by SPA for modeling, the accuracy of the training set was significantly increased compared with the test set by using the PLS and RF methods. When modeling with the band selected by CARS, the model constructed by PLS was more accurate and stable than the model constructed by RF. Similarly, through comparative analysis, we determined that the optimal method for TLA estimation was the CARS-PLS method (Table 3).

### 3.4. Estimation of the Maize SPAD Value

**Figure 7:**
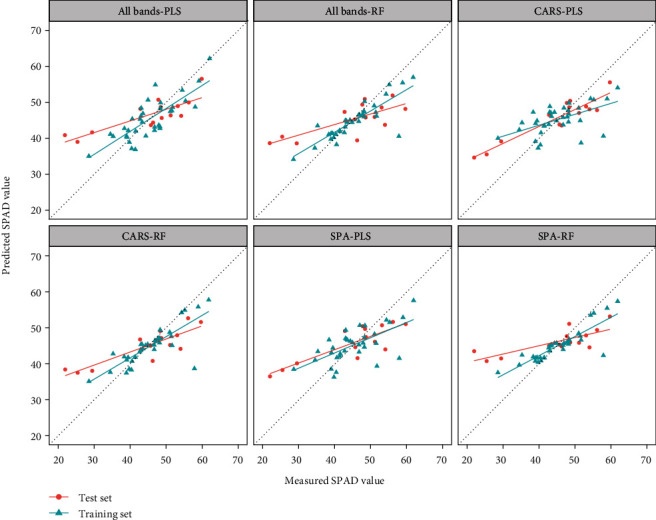
SPAD value prediction using different band combinations and the PLS or RF model.

The sensitive bands for the SPAD value were screened by the SPA and CARS algorithms (Table 4). The bands selected by SPA were 722 nm in red light and 770 nm in near-red light. The correlation coefficient between the 722 nm and 770 nm and the SPAD value were -0.66 and 0.14, respectively. The CARS algorithm selected a wider range of bands, covering a range of 462-726 nm. Table 4 lists the results of the SPAD value model constructed by the PLS and RF methods.  Figure 7 shows a scatter plot of the measured and predicted SPAD value.

On the whole, compared with AGB and TLA, the estimation model of SPAD value had better accuracy and stability. Also, we determined that the optimal estimation method for SPAD value was the combined CARS-PLS (Table 4). It performed best with *R*^2^ of the training set was 0.66, RMSE of 4.95, and MAE of 4.42, and *R*^2^ of the test set was 0.86, RMSE of 6.24, and MAE of 5.11.

### 3.5. TWK Prediction

**Figure 8:**
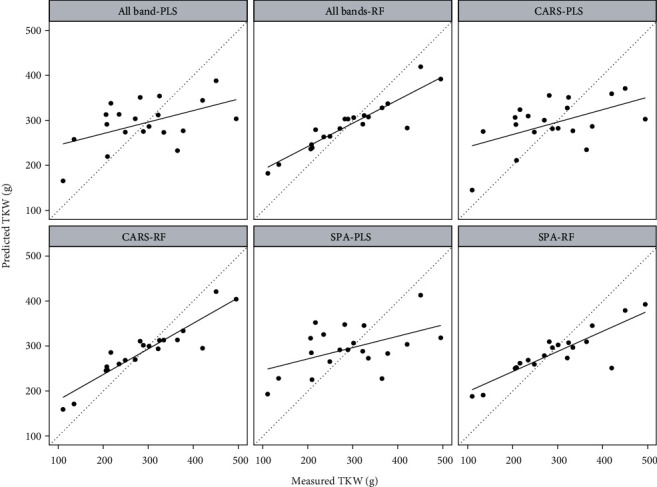
TWK prediction using different band combinations and the PLS or RF model.

The sensitive bands for TWK were screened by the SPA and CARS algorithms (Table 5). There were 8 bands screened by SPA. The correlation coefficients between spectral reflectance of 454-950 nm and TWK were from -0.44 to 0.43. The CARS algorithm selected more bands than SPA, with a total of 50 bands, which were located between 578-718 nm and 874-926 nm, respectively. Table 5 lists the results of the TWK model constructed by the PLS and RF methods.  Figure 8 shows a scatter plot of predicted TWK and the measured TWK.

Regardless of the band combination, the accuracy of the RF model was far higher than that of the PLS model in TWK prediction. Also, we determined that the optimal TWK estimation method was the CARS-RF combination with *R*^2^ value was 0.85, RMSE value of 48.22 g, and MAE value of 38.53 g.

**Figure 9:**
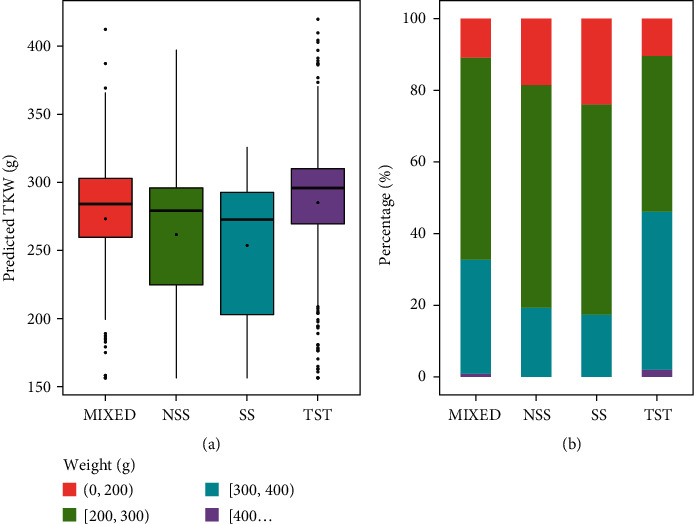
Prediction results of maize TWK based on the optimal combination of the CARS-RF method. Boxplot (a) and percentage chart of the yield levels (b) of different genotypes of materials.

Based on the optimal model of TWK and hyperspectral data, we predicted the TWK of other maize materials. According to the predicted TWK (Figure 9), the distribution interval of TWK on each subgroup genotype was different, indicating that the prediction results are responsive to the laws of materials. It could be seen from Figure 9(a) that the TWK level of TST material was the highest among the four genotypes, with an average TWK of approximately 300 g, and materials between 200 and 400 g accounted for nearly 90% of the total material (Figure 9(b)). However, there were also many outlier values of TST materials, which may be due to the temperate climate of Henan. In temperate zones, the growth period of tropical materials is long, and the yields are not well reflected.

## 4. Discussion

Currently, most physiological and biochemical traits of crops were predicted based on UAV digital or multispectral images to construct vegetation indices [53–55], such as NDVI for AGB, NDRE for chlorophyll content, and RVI for leaf area index. However, in the late stage of crop growth, vegetation indices are so easily saturated that cannot be suitable for estimating crop traits [56]. Considering the grain filling stage of maize, we use the method of selecting sensitive bands instead of vegetation index to estimate the maize traits. The original spectral data could reduce the error propagation and avoid saturation.

Previous researches showed that nonimaging hyperspectral data had good accuracy in estimating crop traits [57–59]. There were relatively few researches on the application of UAV imaging hyperspectral data to estimate crop traits. The applications of UAV imaging were mostly focused on digital and multispectral images. Compared with digital and multispectral images, UAV hyperspectral images have more capability to monitor the subtle features of targets because of their high spatial and spectral resolution [33]. For breeding, the difference of crop traits among varieties is small. It is necessary to obtain multiple traits at the same growth stage in order to comprehensively evaluate varieties. In the study, the UHD185 hyperspectral imager carried by UAV was used to estimate the traits of 498 maize inbred lines, including AGB, TLA, SPAD value, and TWK. By using SPAD value instead of chlorophyll content, the time lag of laboratory detection can be avoided. Although the relationship between SPAD and chlorophyll content is affected to some extent by crop species, growth stages, and environment conditions, it has little effect when applied for small-scale maize inbred lines at the same growth stage. To minimize information redundancy, the SPA and CARS methods were used to screen the most sensitive bands from the full spectra for predicting various traits. The sensitive bands for AGB, TLA, SPAD value, and TWK were all located around 770 nm in the near-red region, which was consistent with previous researches [60–64]. On the whole, compared with the bands selected by the SPA algorithm, CARS could obtain the optimal variable subset through an adaptive weighted sampling technique and exponential decay function. This method had also been applied to the study of the AGB of sugar beets [34]. Our research estimated several common crop traits using the CARS method and obtained similar bands and results. The narrow bands selected by the SPA and CARS algorithms are exclusive compared with the wide bands of multispectral images. The information obtained from wide bands is fused together, which is not conducive to data mining. Narrow bands of hyperspectral image have more subtle and sensitive abilities to capture differences.

The modeling method has a great influence on the estimation accuracy. This study tested two statistical regression methods, PLS and RF. For the AGB, TLA, and SPAD, the model results of the training set and test set for PLS were basically consistent, and the model was robust. The results were consistent with those of Yue et al. [65], who evaluated the advantages and disadvantages of several models in estimating wheat biomass. For the RF model, the results of the training set were better than those of the PLS model. With regard to TWK prediction, the RF model had a much higher accuracy in leave-one-out cross validation than the PLS model. It may be the overfitting of RF model caused by fewer samples. We will further test the overfitting effect of the RF model with more samples. Random forest has a great advantage over other algorithms due to its strong adaptability to datasets, which reflects the advantages of classical machine learning over traditional regression analysis [60, 66].

In breeding, hundreds or even thousands of plots are usually existed in a small area. The efficiency of manual screening of superior varieties is low. In this study, we used the UAV hyperspectral images to realize the estimation of multiple traits of maize inbred lines. It will provide new data sources and technical means for efficiently obtaining field maize phenotypic information and yield predictions and for screening high-yield maize varieties. The estimation accuracy of maize traits in our research was relatively low, which was probably due to the insufficient diversification of experimental data. We also believe that modeling with multiple growing stages data will improve the estimation accuracy. However, the model constructed in this way will produce greater errors when applied for monitoring crop traits in a single growing stage. The information of other growing stages will adversely affect the estimation of single growing stage. In practical application, monitoring maize traits of a specific single growing stage is more useful for breeding. UAV hyperspectral data have been widely used in the agriculture and forestry fields [61, 62], but research on their combination with breeding is still relatively lacking. Many previous studies have focused on a single trait, such as plant height, AGB, or leaf area index. Multiple phenotypic traits need to be comprehensively considered in breeding. Our current research not only considered the population structure traits of AGB and TLA but also explored the nutrition index SPAD value and finally used hyperspectral data to predict the maize TWK for a wide range of maize materials. For tropical and subtropical materials, there are relatively many singular values in TWK prediction, which may be due to the lesser application of related materials in modeling, resulting in the poor representativeness of models.

## 5. Conclusions

In this paper, the UHD185 hyperspectral imager mounted on a UAV was used to obtain hyperspectral images of maize inbred lines in the field. The feasibility of hyperspectral diagnosis of the AGB, TLA, and SPAD value and TWK was studied. A model for quantitatively estimating the AGB, TLA, SPAD value and TWK was constructed, compared, and analyzed using two sensitive band selection methods, SPA and CARS, and two modeling methods, PLS and RF. The major findings are as follows:

1. The band most relevant to AGB was 706 nm (*R* = −0.54). The band most relevant to the SPAD value was 714 nm in the red-edge region (*R* = −0.69). The band most relevant to TLA was 770 nm in the near-red region (*R* = 0.4). Compared with the AGB, TLA, and SPAD value, the correlation between the band at 694 nm and TWK was the best (*R* = −0.5)
2. Compared with SPA on the whole, CARS selected more feature bands that had better accuracies in estimating the AGB, TLA, and SPAD value and TWK. In terms of AGB, TLA, and SPAD value, the optimal method was CARS-PLS. The PLS model had a better prediction accuracy than the RF model based on the entire spectra, bands screened by SPA or CARS. Regarding the maize TWK prediction, the optimal method was CARS-RF, and the RF model performed better than the PLS model. Based on the constructed model, TWK predictions for the remaining materials were conducted. The distribution interval of TWK among the four subgroups was different, indicating that there was a response of the TWK prediction to the laws of the maize materials. Further validation of the TWK prediction for other materials needs to be thoroughly conducted

## Figures and Tables

**Table 1:** Basic statistics of the field measurements.

Date	Period	Object	Min	Max	Mean	CV (%)
2019.9.22	Grain filling stage	AGB (g/plant)	34.08	255	140.39	30.89
		TLA (m^2^)	0.11	0.43	0.3	23.16
		SPAD	21.9	61.77	45.15	18.81

2019.10.24	Harvest time	TKW (g)	110	494.68	289.53	34.5

CV: coefficient variation.

**Table 2:** Results of aboveground biomass (AGB) estimation based on different band combinations and PLS or RF regression.

Bands	Wavelength (nm)	Method	Training set			Test set		
			*R* ^2^	RMSE	MAE	*R* ^2^	RMSE	MAE
All bands	450-950	PLS	0.41^∗∗^	35.67	31.06	0.38^∗^	32.86	25.74
		RF	0.43^∗∗^	40.34	34.80	0.36^∗^	29.74	23.13

SPA	718, 770	PLS	0.57^∗∗^	33.53	29.72	0.26^n.s.^	35.33	28.60
		RF	0.58^∗∗^	38.18	32.39	0.27^n.s.^	33.56	24.94

CARS	486, 510-514, 606, 710-718, 758-770, 894-902	PLS	0.55^∗∗^	31.33	27.53	0.48^∗∗^	28.53	21.23
		RF	0.42^∗∗^	39.33	33.17	0.27^n.s.^	32.37	24.24

n.s., ^∗^, and ^∗∗^ indicate “not significant,” *p* < 0.05, and *p* < 0.01, respectively.

**Table 3:** Results of total leaf area (TLA) estimation based on different band combinations and PLS or RF regression.

Bands	Wavelength (nm)	Method	Training set			Test set		
			*R* ^2^	RMSE	MAE	*R* ^2^	RMSE	MAE
All bands	450-950	PLS	0.54^∗∗^	0.06	0.05	0.18^n.s.^	0.07	0.06
		RF	0.40^∗∗^	0.07	0.05	0.26^n.s.^	0.09	0.07

SPA	770	PLS	0.56^∗∗^	0.06	0.05	0.18^n.s.^	0.08	0.06
		RF	0.48^∗∗^	0.07	0.06	0.06^n.s.^	0.1	0.08

CARS	486, 502, 606, 634, 682, 722, 770-774, 782, 878, 910	PLS	0.73^∗∗^	0.04	0.04	0.62^∗∗^	0.05	0.04
		RF	0.57^∗∗^	0.06	0.05	0.05^n.s.^	0.09	0.07

n.s. and ^∗^ indicate “not significant” and *p* < 0.01, respectively.

**Table 4:** Results of the SPAD estimation based on different band combinations and PLS or RF regression.

Bands	Wavelength (nm)	Method	Training set			Test set		
			*R* ^2^	RMSE	MAE	*R* ^2^	RMSE	MAE
All bands	450-950	PLS	0.65^∗∗^	5.66	4.94	0.68^∗∗^	7.99	6.18
		RF	0.50^∗∗^	4.99	4.39	0.56^∗∗^	8.35	6.82

SPA	722, 770	PLS	0.72^∗∗^	5.03	4.29	0.70^∗∗^	7.51	6.13
		RF	0.61^∗∗^	5.45	4.81	0.59^∗∗^	8.85	6.55

CARS	462, 470-486, 614, 694, 714-718, 726	PLS	0.66^∗∗^	4.95	4.42	0.86^∗∗^	6.24	5.11
		RF	0.52^∗∗^	4.97	4.21	0.74^∗∗^	7.44	5.91

^∗∗^ indicates *p* < 0.01.

**Table 5:** Results of TWK estimation based on different band combinations and PLS or RF regression.

Bands	Wavelength (nm)	Method	*R* ^2^	RMSE	MAE
All bands	450-950	PLS	0.25^∗^	84.57	69.88
		RF	0.84^∗∗^	51.97	40.86

SPA	454, 550, 578, 730, 762, 918, 942, 950	PLS	0.24^∗^	85.19	69.92
		RF	0.75^∗∗^	59.64	46.73

CARS	578-718, 874-926	PLS	0.27^∗^	83.26	67.16
		RF	0.85^∗∗^	48.22	38.53

^∗^ and ^∗∗^ indicate *p* < 0.05 and *p* < 0.01, respectively.

## Data Availability

The data used in this study are freely available. Anyone who wants to use the data can contact the corresponding author Yuntao Ma. The author is with the College of Land Science and Technology, China Agricultural University, Beijing, 100193, China (e-mail: yuntao.ma@cau.edu.cn).
